# New Combinational Assay Using Soluble Fibrin and D-Dimer Determinations: A Promising Strategy for Identifying Patients with Suspected Venous Thromboembolism

**DOI:** 10.1371/journal.pone.0092379

**Published:** 2014-03-24

**Authors:** Shahsoltan Mirshahi, Claudine Soria, Basile Kouchakji, Gérald Kierzek, Jeanne Yvonne Borg, Rémi Varin, Jean Chidiac, Ludovic Drouet, Massoud Mirshahi, Jeannette Soria

**Affiliations:** 1 Service d’Onco-Hématologie, Hôtel-Dieu, Paris, France; 2 Université Paris Diderot Paris-7, UMR INSERM U965, Paris, France; 3 Laboratoire d’Hématologie, Hôpital Lariboisière, Paris, France; 4 Laboratoire MERCI, Faculté de Médecine et Pharmacie, Rouen, France; 5 Service de Pneumologie, Hôpital de Neuilly, Neuilly, France; 6 Service des Urgences, Hôtel-Dieu, Paris, France; 7 Hôpital Charles-Nicolle, Rouen, France; Medical University Hamburg, University Heart Center, Germany

## Abstract

**Aim:**

To establish a new and reliable assay for quantification of the soluble fibrin (SF) in combination with that of D-dimer for early diagnosis of venous thromboembolism.

**Methods and Samples:**

The SF assay is based on D-dimer generated after incubation of plasma with tissue-type plasminogen activator (t-PA). SF and standard D-dimer assays, run in blind, were used to test 119 untreated outpatients with clinically suspected deep-vein thrombosis (DVT, 49 patients) or pulmonary embolism (PE, 70 patients) consulting at the emergency unit of the hospital. Thromboses were confirmed by current imaging methods such as ultrasonography, scintigraphy, computed tomographic pulmonary angiography (CTPA) and ventilation/perfusion scan.

**Results:**

SF assay was validated in 270 healthy volunteers [51.8% males; mean age years ± SD: 41±13; age range 19 to 65]. Among these normal plasmas, SF levels were ≤200 ng/mL in 97.8% of them, and 200–250 ng/mL in the remainder [26–46 years old; 50% males]. ROC curves were used to determine the SF cut-off value for plasma SF positivity, which was found to be 300 ng/mL. In patients with suspected venous thromboembolism, SF sensitivities for DVT and PE (92% and 94%, respectively) were comparable to those of D-dimer (96% and 94%), whereas SF specificities (86% and 95%) were higher than those of D-dimer (50% and 54%). Positive-predictive values for SF (89% and 94%) were again higher than those of D-dimer (70% and 65%) in DVT and PE. The amount of circulating SF normalized rapidly after anticoagulant therapy.

**Conclusion:**

Results from this small group of patients suggest that the evaluation of plasma SF, in combination with that of D-dimer, represents a potentially useful tool for the early diagnosis of venous thromboembolism, provided that the patients have not been treated previously by anticoagulants.

## Introduction

Venous thromboembolism (VTE), which includes deep-vein thrombosis (DVT) and pulmonary embolism (PE), is the third most common cardiovascular disease after acute coronary syndrome and stroke [1]. It is due to a combination of hereditary and/or acquired risk factors such as vessel-wall damage, venous stasis and increased activation of clotting factors [2].

Plasma D-dimer (fibrin-degradation products) measurement provides information about fibrin formation followed by fibrinolysis. It is currently used to exclude the diagnosis of VTE because of its excellent negative-predictive value (NPV) [3], [4]. However, an elevated D-dimer concentration alone does not confirm DVT diagnosis and cannot be used for its positive-predictive value (PPV) [5], [6], since increased D-dimer levels can also be detected in patients with malignancy, trauma, recent surgery, infection and active bleeding [7], [8]. It was previously reported that low soluble fibrin (SF) concentrations are detected in normal plasma, and that high concentrations are found in patients with thrombotic disease, especially in the early stages [9], [10]. SF composition is heterogeneous and depends on the degree of fibrin-monomer polymerization [11]. Despite the low number of fibrin monomers in SF, they are cross-linked together by activated factor XIII, whose activation coincides with fibrinopeptide A (fpA) release [12].

Among the available biomarkers for the pre-thrombotic state, *in vivo* thrombin generation has been evaluated by fpA quantification (half-life 3 min) [13] or thrombin–antithrombin complex formation (half-life 15 min) [14]. We chose to study the biomarker SF in plasma due to the fact that it has a longer circulating half-life (2–6 h) [15], [16]. Because of this characteristic, several assays have been developed to evaluate SF in the plasma of patients with suspected thrombosis, but the high variability of results obtained with different assays has thus far prevented the use of SF levels for diagnosing VTE. The aim of this study was to evaluate the potential usefulness of a new plasma SF assay for diagnosing suspected thromboembolic events. Developed in our laboratory, this technique is based on the *in vitro* generation of D-dimer from soluble fibrin during the incubation of plasma with tissue plasminogen activator (t-PA).

## Material and Methods

### Healthy volunteers

The volunteers were from the blood bank of Hôtel Dieu de Paris (Agreement with INSERM) [males  =  51.8%, females  =  48.2%, age ranging from 19 to 65 years].

### Patients

This study was conducted in accordance with the Helsinki Declaration of 1975. The measurements were made on patients’ blood left over after routine analyses had been run, in accordance with the French law for biomedical research (Code de la Santé Publique. Article L1211-2, modified by the law n°2004–800 of August 6th, 2004 - published in: art. 7 in Journal Officiel de la République Française of August 7th, 2004). The plasmas were provided to us as anonymous samples identified only by the age and sex of the patients, along with their diagnosis, i.e., DVT/PE + or –, and heparin treatment status (+ or –). Specimens used were from patients having routine coagulation investigated in several hospital laboratories (Lariboisière Hospital, Paris; Neuilly Hospital; Charles-Nicolle Hospital, Rouen; Hôtel Dieu, Paris). Blood samples tested were those collected in tubes containing 0.105 M citrate (1 part citrate to 9 parts blood). Plasmas were obtained by blood centrifugation at *2,500 g* for 15 min, then collected and stored at –20°C until use. A sample once thawed was never refrozen. After thawing samples (healthy volunteers from blood bank or patient samples), we always checked that there was no clot in the tube.

In massive disseminated intravascular coagulation (DIC), SF may precipitate as an insoluble complex during freezing and thawing; it is therefore recommended, for suspicion of DIC, that this assay be run on freshly collected plasma.

In this pilot study, 119 untreated outpatients, recruited in a multi-centric study among patients examined in emergency departments and presenting with clinical symptoms of DVT or PE, were eligible to participate. Imaging methods used for diagnosis of PE were scintigraphy, computed tomographic pulmonary angiography (CTPA) and ventilation/perfusion scan, and that used for DVT diagnosis was compression ultrasonography. Ongoing anticoagulant therapy was the only exclusion criterion. SF and D-dimer were measured in all 119 patients [53.7%, male, 46.3% female; mean age (± SD) 61±19 years; age range, 28–93] at the time of hospital admission, before administration of any anticoagulant. Those who had previously been treated with unfractionated or low-molecular-weight heparin were not enrolled in this study and were considered only for follow-up. Among the 49 patients with suspected DVT, a thrombosis was visualized by ultrasonography for 27 [53.3% male, 46.7% female; mean age (± SD), 67±17 years; age range, 37–93], whereas the diagnosis was rejected for the remaining 22 patients. Among the 70 patients with suspected PE, 33 were confirmed by imaging [46.1% males, 53.9% females; mean age (± SD), 60±21; age range, 28–91], whereas this diagnosis was not retained for the other 37 patients.

### Tissue plasminogen activator (t-PA)

The t-PA used in our assay was Actilyse (Boehringer, Ingelheim).*Thrombin* was of human origin, from the fibrin sealant kit “TISSEL*”* (Baxter, Braine-l’Alleud) used therapeutically to stop bleeding. *Aprotinin* (10,000 K. I.U./mL) was from Bayer (Trasylol, kindly given by Bayer, Puteaux, France, not for clinical use). *Fibrin-degradation products* were obtained by adding 0.5 mL of CaCl_2_ M/40, 0.2 mL of t-PA at 100 μg/mL and 0.2 mL of thrombin at 50 I.U./mL to 0.5 mL of normal plasma. Under these conditions, a clot occurs in few seconds. After clot formation, plasmin is generated, inducing the complete degradation of the fibrin network, leading to the production of soluble fibrin degradation products within less than 30 min. After complete degradation of the clot (no residual clot was visible in the tube), plasmin generated was blocked by adding 0.3 mL of aprotinin at 10,000 K.I.U/mL.

### Biological assays

#### 1. D-dimer levels were measured by agglutination of latex-coated microparticles with monoclonal antibodies directed against D-dimer using STA-Liatest D-DI (Stago, Asnières, France) on an STA apparatus and were expressed in fibrin equivalents

Using this technique, it was shown that the within-run precision estimates (coefficient of variation) at mean D-dimer levels of 170 ng/mL (170 μg/) and 2,400 ng/mL (2,400 μg/L) were 19.2% and 2.9%, respectively [17].

#### 2. The SF assay was performed in 3 steps

*First:* SF degradation into D-dimer: to 200 μL of plasma was added either 20 μL of t-PA at 20 μg/mL (treated plasma) or 20 μL of saline (untreated plasma). After a 15-min incubation at 37°C, the plasmin generated was blocked by the addition of 20 μL of aprotinin previously diluted 1/25 in 0.15 M NaCl. After each reagent was added, the tube shaken manually to ensure thorough mixing.

*Second:* Determination of D-dimer concentration in treated and untreated plasma.

*Third:* SF concentrations, represented by D-dimer generation in plasma treated by t-PA, were calculated as the difference between D-dimer concentrations found in tPA-treated plasma and in untreated plasma. Indeed, the results can be expressed in fibrin equivalents, since D-Dimer levels are already expressed in fibrin equivalents.

When the plasma D-dimer level exceeded 4000 ng/mL, the sample was diluted after the degradation step. It should be pointed out that, in preliminary assays, we determined the optimum t-PA concentration and incubation time of plasma with t-PA to be used, i.e., appropriate conditions such that 1) plasma fibrinogen (whatever its level) was not degraded, and 2) the SF level was dependent on the thrombin concentration.

The positive control, which is prerequisite for this assay, was an SF-enriched plasma kindly provided by Stago (prepared using a special undisclosed procedure, and for which the concentration was determined spectrophotometrically at 280 nm using the extinction coefficient reported in the literature, [18]. An aliquot of purified SF was diluted in normal plasma, and the concentration of SF determined by evaluating the difference between D-dimer concentration before and after t-PA addition. Then the concentration of SF was adjusted to obtain a concentration of 2,000 ng/mL. The negative control was the same plasma, but with no SF added. The positive and negative controls were aliquoted and the samples freeze-dried. Before use, these freeze-dried control samples were reconstituted with distilled water and kept at room temperature (18–25°C) for 30 min followed by swirling of the vial. If reagents from other suppliers were to be used, the conditions necessary for determining the threshold levels for positivity would of course have to be determined experimentally.

### Repeatability and reproducibility of the test using appropriate controls

The repeatability of the test was evaluated both by 10 successive determinations of SF level in positive controls obtained by adding various amounts of purified SF (Stago) to a normal plasma immediately before testing, i.e., without freeze drying; and by 21 successive determinations on a freeze-dried positive control from Stago. The reproducibility of the test was evaluated by 12 determinations carried out on the freeze-dried positive controls containing 2,000 ng/mL of SF. The samples were stored at 4°C, and, on day 1 and day 30, 12 aliquots were tested in the SF assay. The day-to-day reproducibility was determined by a single determination of SF in positive control plasma per day during 30 days. The long term stability of the freeze-dried positive controls from Stago was determined both: i) after a 4 week thermic stress at 30°C, calculated by the median relative difference of SF levels between samples kept at +2°C to +8°C and those kept at +30°C and ii) after 5 months at 2°C to 8°C, evaluated as the median relative difference of SF value between the samples at day 1 and those kept for 5 months.

### Determination of the cut-off value for SF positivity

Receiver operating characteristic (ROC) curves, generated using XL-Stat software, were used to determine the cut-off value for plasma SF that minimizes the number of false positives and false negatives. Minimizing the false positives and false negatives is equivalent to maximizing sensitivity and specificity.

ROC curves were performed using plasma SF (or D-dimer) concentrations in all patients tested for suspicion of thromboembolic disease. Knowledge of the presence or absence of thromboembolic disease, evaluated by imaging, was used to create a ROC curve for the SF (or D-dimer) assay, the levels of SF (or D-dimer) being classified according to the presence or absence of thromboembolic disease. The ROC curve was obtained [Y-axis  =  the true positive rate (Sensitivity) and X-axis  =  the false positive rate (1-Specificity)], and the area under the curve (AUC) was evaluated [19]. The cut-off was determined by evaluation of both:

1) The location of the value that minimizes the Euclidean distance between the ROC curve and the upper left corner of the graph: this value point is determined exactly, by referring to the ROC Analysis tables (Appendices).

2) The value that maximizes the sum of sensitivity and specificity. The optimal threshold point is to identify the best cut-off that maximizes (Sensitivity + Specificity). In the figures, XL-Stat plots show, in the same graph, two curves: Sensitivity (as a function of Concentration) and Specificity (as a function of Concentration). The optimal cut-off is the point where the two curves intersect.

### Statistical analyses

SF and D-dimer levels are reported as means ± 1 standard deviation (SD) and as median (25th and 75th percentiles). The non-parametric Wilcoxon signed-rank test was used to compare the SF or D-dimer concentrations of patients with or without PE and/or DVT. Sensitivity, specificity, negative-predictive values (NPV) and positive-predictive values (PPV) and their 95% confidence intervals (CI) for VTE diagnosis were calculated using SAS v9.2 software (SAS Institute Inc., Cary, NC). Tests were two-sided, and a *p* value <0.05 was considered to be statistically significant. To compare the performance characteristics of SF with that of D-dimer for diagnosis of venous thromboembolism [19], the area under the ROC curve (AUC), an important measure of the accuracy of the clinical test, was determined. The diagnostic value of SF, compared to D-dimer using AUC, is determined following the procedure of Hanley and McNeil [20], using XLSTAT Software.

## Results

### Validity of the assay

#### Assay specificity

*Absence of interference of fibrin degradation products.* Fibrin-degradation products added in very high amounts (3,750–15,000 ng/mL) to normal plasma did not induce any increase in the SF concentration, demonstrating that there was no interference by fibrin degradation products (up to 15,000 ng/mL) (Table 1).

**Table 1:** Specificity of the test: Absence of interference by fibrin degradation products.

Amount of D-dimer added to normal plasma (ng/mL)	Basal value of plasma D-dimer (ng/mL)	Plasma value of plasma D-dimer after t-PA addition (ng/mL)	Mean value of plasma SF (ng/mL)
**0**	210*/210*	210*/210*	≤ 200**
**3,750**	**3,820/3,930**	**3,950/4,000**	**100**
**7,500**	**7,820/7,990**	**7,990/7,730**	**0**
**15,000**	**15,830/15,350**	**15,730/15,460**	**5**

(*) 210 ng/mL is the detection limit of the STA Liatest.

(**) the levels of D-dimer in untreated plasmas and in t-PA treated plasmas being beneath the detection limit of D-dimer (i.e., 210 ng/mL), we conclude only that the SF concentration in this group was ≤200 ng/mL.

Fibrin degradation products (D-dimer) were added to normal plasma at the indicated final concentrations. SF was then determined.

For each sample, D-dimer measurement was performed in duplicate (but this is not strictly necessary).

It has to be pointed out that large amounts of D-dimer were added to normal plasma (from 3,750 to 15,000 ng/mL) because these concentrations of plasma D-dimer were often observed in pathological clinical samples. In this test, the CV of D-dimer determination is between 1.4% and 2%, in good correlation with those reported by others using the same technique as that used here for SF evaluation (Lia-test): indeed, the D-dimer coefficient of variation (CV) depends on D-dimer concentration; and at mean levels of 170 ng/mL and 2,400 ng/mL the reported CV were 19.2% and 2.9%, respectively [17].

*Absence of interference of fibrinogen levels present in plasma.* The SF assay was performed using plasmas from normal volunteers in whom the fibrinogen levels varied from 2 g/L to 5 g/. In these plasmas, no SF was detectable, demonstrating that high levels of fibrinogen do not increase the measured SF level.

*Absence of interference of t-PA present in plasma.* The levels of t-PA in normal plasmas and in patients’ plasmas are known to be much lower (about 1000-fold less) than that used in the SF test (2000 ng/mL) and therefore do not interfere with the test, since it was reported in the literature that in patients with PE, the plasma t-PA level was 21.3±7.5 ng/mL (i.e., 14.06±4.95 IU/mL) versus 15.2±10 ng/mL (i.e., 10±6.6 IU/mL) in patients negative for PE and 3±1.5 ng/mL (i.e., 1.98±1 IU/mL) in controls [21], [22]. In patients with DVT, the plasma t-PA level was 11.4±4.4 ng/mL (i.e., 7.52±2.9 IU/ml) versus 9.4±4.1 ng/mL (i.e., 6.2±2.7 IU/ml) for controls [23].

*Absence of interference of PAI present in plasma.* It has been reported in the literature that in patients with PE, the plasma PAI-1 level was 36.9 ±13 ng/mL (i.e., 33.13±11.67 AU/mL) versus 27.4±14 ng/mL (24.6±12.57 AU/mL) in PE negative patients and 19 ±13 ng/mL (17.06±11.6 AU/mL) in controls [21].

In patients with DVT, the plasma PAI-1 level was 20.27 ±14 ng/mL (i.e., 18.2±12.6 AU/mL) versus 19.49±11.8 ng/mL (i.e., 17.5±10.6 AU/mL) in controls [23].

Since 1 AU of active PAI-1 inhibits 1 IU of active t-PA [21], it can be deduced that there is no residual t-PA activity in any of our plasma samples (whether from patients or from normal controls), and that the residual activity of PAI-1 was always less than 15 AU/mL. Therefore, the concentration of t-PA used in the test is greatly in excess, compared to PA and PAI levels.

#### Repeatability and Reproducibility of the test

*Repeatability of the test.* Evaluation by 10 determinations of SF in 8 positive controls containing various levels of SF (from 859 to 1,797 ng/mL):

In these 8 positive controls, the mean levels of SF were 859, 1,098, 1,186, 1,216, 1,465, 1,620, 1,666 and 1,797 ng/mL, respectively, and the intra-assay CVs, calculated according to the CLSI protocol, were 10.73**%,** 6.75%, 7.62%, 5.85%, 7.75 %, 2.45%, 3.55 % and 4.3 %, respectively. In the 2 controls containing a mean level of SF near the cut-off value (276 ng/mL and 327 ng/mL), the intra-assay CVs were 8.2% and 8.4%, respectively. In one negative control containing 135 ng/mL of SF, the intra-assay CV was 4.8%. Taking into account all these results (high and normal levels of SF), the intra-assay CV of SF determination was found to be 6.4±2.46 %. Evaluation by successive determinations on the freeze-dried positive control from Stago: 21 aliquots of the same batch were tested. The mean coefficient value ± SD was 1,860±100 ng/mL and the intra-assay CV value was 5.27%.

*Reproducibility of the test.* From this same batch of freeze-dried positive control plasma, 12 additional aliquots were tested after 30 days storage at +2°C to +8°C. The mean SF concentration was found to be 1,750±140 ng/mL in samples kept for 30 days (versus 1,860±100 ng/mL for day 1, as explained above), and the corresponding CV was 8.02 %.

For the day-to-day reproducibility, the mean level of SF in freeze-dried SF-spiked plasma was found to be 1,750±100 ng/mL, and the calculated coefficient of reproducibility was 7%.

*Long term stability of the freeze-dried positive controls.* The 4 week thermic stress stability, calculated by the median relative difference of SF levels between samples kept at +30°C and those kept at +2°C to +8°C was 4.0%, suggesting a long-term stability equivalent to 2 years. The long-term stability at +2°C to +8°C, calculated by the median relative difference between SF levels at day 1 was +2.5% after 5 months.

### Investigations for diagnosis of venous thromboembolism

#### 1. Normal values

For determining normal values, SF was evaluated in 270 normal plasmas from healthy volunteers aged 19 to 65 years. In 240 of them (52.1% males) from 19 to 65 years old, the levels of D-dimer in untreated plasmas and in t-PA treated plasmas were beneath the detection limit of D-dimer (i.e., 210 ng/mL), hence we can conclude only that the SF concentration in this group of patients was ≤200 ng/mL. In the 30 other normal volunteers (50% males) the levels of D-dimer in untreated and t-PA treated plasmas being above the threshold value, the estimated calculated concentrations of SF were between 50 and 250 ng/mL: SF levels of 50–200 ng/mL were found in 24 volunteers, 32 to 65 years old (50% males); and SF levels between 200 and 250 ng/mL were found in 6 healthy volunteers, 26 to 46 years old (2.2% of the tested population, 50% males). The cut-off value of 300 ng/mL was chosen using the ROC analysis (see above, *SF and D-dimer levels in patients with suspected PE and/or DVT* ***-*** *Evaluation of the cut-off value for SF*).

#### 2. Decrease of plasma SF level after starting anticoagulant therapy

**Figure 1:**
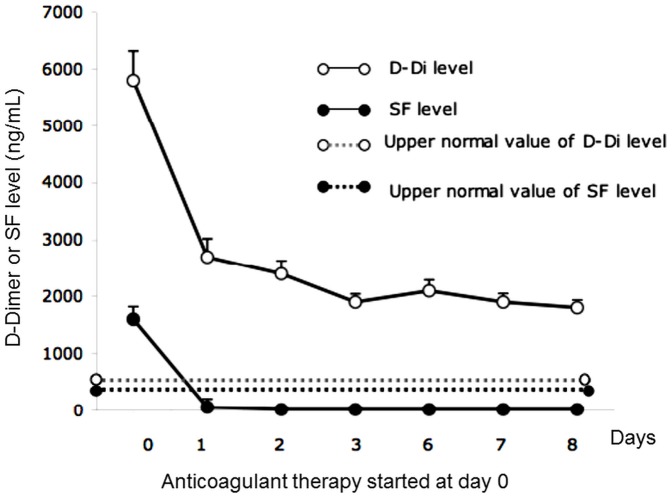
Evolution of SF and D-dimer levels in patients under anticoagulant therapy. SF and D-dimer (D-Di) concentrations were determined daily in patients before and after starting anticoagulant therapy.

The modification of SF concentration in patients under heparin therapy was analysed. As presented in Figure 1, the SF concentrations dropped rapidly after starting anticoagulant therapy, which was consistent with the 2–6 h SF half-life [16], while D-dimer levels remained elevated for several days until the clot was completely degraded. A day after starting anticoagulant therapy, SF levels returned to normal or were at the upper limit of normal. Analysis revealed that, under heparin treatment, the SF values (performed daily) remained within the normal range (Figure 1). Only 1 patient’s SF level increased when therapy was stopped. In contrast, D-dimer levels declined only slowly and did not become normal within the first 8 days after starting anticoagulant treatment.

#### 3. SF and D-dimer levels in patients with suspected PE and/or DVT

**Figure 2:**
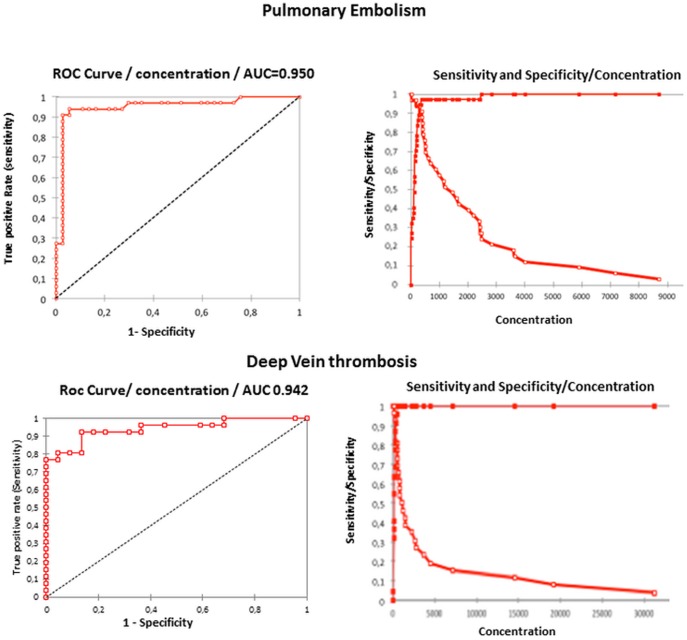
ROC curves for DVT and PE. ROC Curves established for Pulmonary Embolism and for Deep Vein Thrombosis Open squares : Sensitivity Closed square : Specificity.

*Evaluation of the cut-off value for SF.* For evaluation of the cut-off point for plasma SF, ROC curves were established using SF levels of patients and an independent diagnosis (imaging) that classified the patients into two distinct groups: a diseased and a non-diseased group. On these ROC curves, we determined 1) the location of the value that minimizes the Euclidean distance between the ROC curve and the upper left corner of the graph, value determined exactly by referring to the ROC Analysis tables and 2) the value that maximizes the sum of Sensitivity and Specificity (Roc appendices in Table 2, ROC curves in Figure 2).

**Table 2:** Roc appendices for pulmonary embolism and for deep vein thrombosis.

Pulmonary embolism					Deep vein thrombosis							
Concentration	Sensitivity	Specificity	PPV	NPV	Concentration	Sensitivity		Specificity		PPV	NPV	
10	1,000	0,000	0,471		10	1,000		0		0,542		
20	1,000	0,243	0,541	1,000	20	1,000		0,045		0,553	1,000	
30	0,970	0,270	0,542	0,909	60	1,000		0,318		0,643	1,000	
40	0,970	0,324	0,561	0,923	80	0,962		0,318		0,625	0,875	
75	0,970	0,351	0,571	0,929	110	0,962		0,364		0,641	0,889	
100	0,970	0,378	0,582	0,933	130	0,962		0,409		0,658	0,900	
110	0,970	0,405	0,593	0,938	140	0,962		0,545		0,714	0,923	
120	0,970	0,486	0,627	0,947	180	0,962		0,636		0,758	0,933	
130	0,970	0,541	0,653	0,952	230	0,923		0,636		0,75	0,875	
140	0,970	0,568	0,667	0,955	270	0,923		0,682		0,774	0,882	
150	0,970	0,649	0,711	0,960	280	0,923		0,773		0,828	0,895	
160	0,970	0,676	0,727	0,962	290	0,923		0,818		0,857	0,900	
180	0,970	0,703	0,744	0,963	**300**	**0,923**		**0,864**		**0,889**	**0,905**	
200	0,939	0,730	0,756	0,931	310	0,808		0,864		0,875	0,792	
210	0,939	0,757	0,775	0,933	400	0,808		0,909		0,913	0,8	
220	0,939	0,784	0,795	0,935	460	0,808		0,955		0,955	0,808	
240	0,939	0,838	0,838	0,939	470	0,769		0,955		0,952	0,778	
260	0,939	0,865	0,861	0,941	490	0,769		1,000		1,000	0,786	
290	0,939	0,892	0,886	0,943	500	0,731		1,000		1,000	0,759	
**300**	**0,939**	**0,946**	**0,939**	**0,946**	520	0,692		1,000		1,000	0,733	
350	0,909	0,946	0,938	0,921	710	0,654		1,000		1,000	0,71	
390	0,909	0,973	0,968	0,923	730	0,615		1,000		1,000	0,688	
395	0,879	0,973	0,967	0,900	760	0,577		1,000		1,000	0,667	
410	0,848	0,973	0,966	0,878	780	0,538		1,000		1,000	0,647	
430	0,788	0,973	0,963	0,837	1 000	0,5		1,000		1,000	0,629	
490	0,758	0,973	0,962	0,818	1 190	0,462		1,000		1,000	0,611	
507	0,727	0,973	0,960	0,800	1 400	0,423		1,000		1,000	0,595	
520	0,697	0,973	0,958	0,783	1 446	0,385		1,000		1,000	0,579	
610	0,667	0,973	0,957	0,766	2 250	0,346		1,000		1,000	0,564	
690	0,636	0,973	0,955	0,750	2 600	0,308		1,000		1,000	0,55	
870	0,606	0,973	0,952	0,735	2 820	0,269		1,000		1,000	0,537	
1000	0,576	0,973	0,950	0,720	3 640	0,231		1,000		1,000	0,524	
1160	0,545	0,973	0,947	0,706	4 500	1,192		1,000		1,000	0,512	
1190	0,515	0,973	0,944	0,692	7 170	0,154		1,000		1,000	0,500	
1446	0,485	0,973	0,941	0,679	14 490	0,115		1,000		1,000	0,489	
1600	0,455	0,973	0,938	0,667	19 250	0,077		1,000		1,000	0,478	
1680	0,424	0,973	0,933	0,655	31 250	0,038		1,000		1,000	0,468	
2020	0,394	0,973	0,929	0,643								
2200	0,364	0,973	0,923	0,632								
2410	0,333	0,973	0,917	0,621								
2420	0,303	0,973	0,909	0,610								
2430	0,273	0,973	0,900	0,600								
2470	0,273	1,000	1,000	0,607								
2480	0,242	1,000	1,000	0,597								
2840	0,212	1,000	1,000	0,587								
3600	0,182	1,000	1,000	0,578								
4000	0,121	1,000	1,000	0,561								
5900	0,091	1,000	1,000	0,552								
7170	0,061	1,000	1,000	0,544								
8690	0,030	1,000	1,000	0,536								

Using these 2 parameters, it was found that the optimal SF cut-off for both PE and DVT disease is 300 ng/mL. The test is positive if the SF concentration is greater than the optimal cut-off value.

*Evaluation of SF and D-dimer plasma concentrations in patients with suspicion of DVT and PE.* D-dimer levels were below the cut-off value in 12 of the 49 patients with suspected DVT and in 22 of the 70 patients suspected of having a PE, whereas their SF levels were below the threshold value in 21 of them for DVT and 37 for PE. Differences between the performances of SF and D-dimer in PE and DVT are presented in Table 3. In patients with suspected PE, it was observed that mean plasma SF and D-dimer levels in the 33 patients with confirmed PE were significantly higher than those of the 37 patients for whom a diagnosis of thromboembolism was rejected (Table 3).

**Table 3:** Plasma Soluble Fibrin (SF) and D-dimer levels in Patients with suspected Pulmonary Embolism (PE) or Deep Vein Thrombosis (DVT).

Parameter	Suspected PE			Suspected DVT		
	PE confirmed	PE rejected	p value	DVT confirmed	DVT rejected	p value
n	33	37		27	22	
**SF (ng/mL), mean ± SD**	**1945±2051**	**181±392**	**<0.01**	**5249±8417**	**178±207**	**<0.01**
**SF ≥ 300 ng/mL, n (%) of patients)**	**31 (94%)**	**2 (6%)**	**<0.01**	**25 (93%)**	**3 (14%)**	**<0.01**
**D-dimer (ng/mL), mean ± SD**	**5530±7751**	**1055±1732**	**< 0.01**	**6966±9086**	**830±730**	**<0.01**
**D-dimer ≥ 500 ng/mL, n (%) of patients)**	**31 (94%)**	**17 (46%)**	**<0.01**	**26 (96%)**	**11 (50%)**	**<0.01**

SF threshold value  =  300 ng/mL; D-dimer threshold value  =  500 ng/mL.

**Figure 3:**
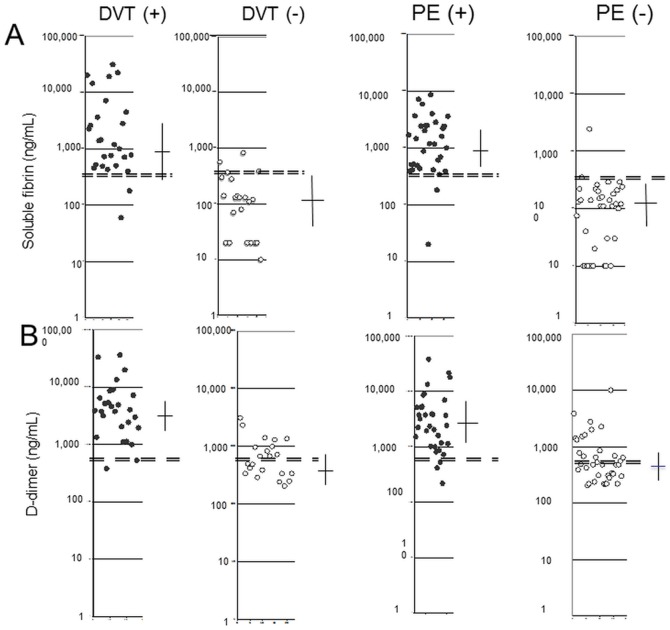
Distribution of the levels of SF and D-dimer in patients with suspected pulmonary embolism (PE) or deep vein thrombosis. Evaluation of median and 25th and 75th percentiles. (A) Soluble fibrin concentration. (B) D-dimer concentration. •/Closed circles: patients with PE or DVT; ○/open circles, patients without PE or DVT. The bars depict the median with interquartile ranges. The double broken bars represent the upper normal value.

Among those 33 patients, 31 had plasma SF and D-dimer concentrations above normal values, whereas 2 patients (false-negative values) had normal SF concentrations (1 with a small PE), but the D-dimer concentration in these 2 patients was high. Two other patients had normal D-dimer concentrations but their SF levels exceeded the 300 ng/mL cut-off value (412 and 340 ng/mL). Among the 37 patients for whom a PE diagnosis was excluded, only 2 had SF levels >300 ng/mL, whereas 17 had D-dimer levels >500 ng/mL (false-positive values) (Table 3). In patients with suspected DVT, we noticed that the mean plasma SF and D-dimer levels (27 patients with confirmed DVT) were significantly higher (29- and 8-fold, respectively) than those of the 22 other patients for whom the diagnosis was excluded (Table 3). Among the 27 patients with confirmed DVT, 25 had high SF levels, whereas the other 2 (false-negative values) had SF concentrations within the normal range. These last 2 patients had plasma D-dimer levels exceeding the threshold value (2440 and 530 ng/mL). Twenty-six of these patients with confirmed DVT had D-dimer concentrations >500 ng/mL and the last had a normal level (false-negative). However, in this patient, the SF level was exceptionally high (1400 ng/mL), perhaps suggesting a failure of fibrinolysis. Among the 22 patients for whom the DVT diagnosis was excluded by Doppler ultrasonography, only 3 had SF levels above 300 ng/mL (false-positive values), the remaining 19 patients’ SF concentrations were within the normal range. Half of these patients had elevated D-dimer levels, and 2 of them had plasma SF levels above the cut-off value (Table 3). As indicated in Figure 3, the SF concentrations were high in the DVT and PE groups, compared to the non-DVT and non-PE groups: the medians (25^th^ and 75^th^ percentiles) for SF and for D-dimer are presented for DVT and non-DVT groups and for PE and non-PE groups.

For SF concentrations, the medians (25^th^ and 75^th^ percentiles) were respectively:

1190 ng/mL (510 – 4,070) in the DVT group, 125 ng/mL (20 – 237) in the non-DVT group

1190 ng/mL (490 – 2,470) in the PE group, 120 ng/mL (20 – 200) in the non-PE group.

For D-dimer concentrations, the medians (25^th^ and 75^th^ percentiles) were respectively:

3900 ng/mL (2,005 – 6,841) in the DVT group, 590 ng/mL (340 – 992) in the non-DVT group, 2420 ng/mL (1,120 – 5,219) in the PE, 480 ng/mL (310 – 860) in the non-PE group.

#### 4. Comparison of the Diagnostic Performances of SF and D-dimer determinations

*Classification of patients as true negative, false negative, true positive and false positive.* It is clearly evident in the results presented in Figure 3 that in patients who presented a confirmed diagnosis of DVT or PE, there are few patients with false negative values for SF or D-dimer, whereas for patients for whom the diagnosis of DVT or PE was excluded, the number of patients with false positive results is more important for D-dimer than for SF levels.

*Performance of SF and D-dimer determinations for diagnosis of venous thromboembolism.* Area under the curve. The comparison was done by evaluating the areas under the ROC curves derived from the same set of patients. The area under the ROC curve (AUC) is a widely used performance score, as it is well established that the best method is the one with the largest AUC [19]. The results of the AUC for SF and for D-dimer derived from patients with a suspicion of DVT and PE are presented in Table 4; they demonstrate that SF is a better biological marker of thromboembolic disease than D-dimer, the difference of AUC between D-dimer and SF being significant (p<0.01 for PE and p = 0.017 for DVT).

**Table 4:** Evaluation of the area under the ROC (Receiving Operating Characteristic) curve for SF and D-dimer derived from patients with suspected Pulmonary Embolism or Deep Vein Thrombosis.

Parameter	Pulmonary embolism		Deep vein thrombosis	
	**SF**	**D-dimer p**	**SF**	**D-dimer p**
**AUC**	**0.950**	**0.846 <0.01**	**0.942**	**0.913 0.017**

*Accuracy indexes.* The accuracy indices (sensitivity, specificity, positive predictive value and negative predictive value, and their respective 95% confidence intervals) were calculated for D-dimer and SF, as described in the Methods section. As shown in Table 5, in patients with suspected venous thromboembolism (DVT n = 49) and (PE n = 70), SF sensitivities (92% and 94%) were similar to those of D-dimer (96% and 94%) respectively, whereas SF specificities (86% and 95%) were higher than those of D-dimer (50% and 54%). Positive-predictive values for SF (89% and 94%) were again higher than those of D-dimer (70% and 65%) in both DVT and PE. These results showed that SF measurement, in combination with that of D-dimer, constitutes a valid approach for predicting probable onset of thromboembolism.

**Table 5:** Sensitivity, Specificity, Positive- (PPV) and Negative-Predictive Values (NPV) (95% Confidence Intervals) for Soluble Fibrin (SF) and D-dimer concentrations in Pulmonary Embolism and Deep Vein Thrombosis.

Parameter	Pulmonary embolism			Deep vein thrombosis		
	SF	D-dimer	p	SF	D-dimer	p
**Sensitivity**	**0.94 (0.86**–**1)**	**0.94 (0.86**–**1)**	**NS**	**0.92 (0.83**–**1)**	**0.96**	**NS**
**Specificity**	**0.95 (0,87**–**1)**	**0.54 (0,38**–**0,7)**	**<0.01**	**0.86 (0.66**–**0.98)**	**0.5 (0.29**–**0.7)**	**<0.01**
**PPV**	**0.94 (0,86**–**1)**	**0.65(0.51**–**0.78)**	**<0.01**	**0.89 (0.74**–**0.99)**	**0.70 (0.56**–**0,85)**	**<0.01**
**NPV**	**0.95 (0.87**–**1)**	**0.91 (0.79**–**1)**	**NS**	**0.90 (0.77**–**1)**	**0.92 (0.76**–**1)**	**NS**

## Discussion

A non-invasive and highly accurate diagnostic tool for DVT and/or PE is needed that would allow immediate treatment decisions to be made for most patients. The objective of the present study was to determine whether the combination of SF and D-dimer measurements could help VTE diagnosis and also monitor the efficacy of anticoagulants in inhibiting thrombogenesis.

It is well established that the D-dimer assay has a poor specificity for the diagnosis of VTE, due to the degradation of extravascular fibrin into D-dimer by local fibrinolytic enzymes, which, because of their low molecular weight, easily diffuse into the bloodstream. This notion is supported by the often elevated D-dimer levels seen in patients with cancer or acute inflammatory diseases [24], [25]. Previous observations showed that D-dimer has a high negative predictive value for excluding venous thromboembolism, but that the positive predictive value of D-dimer for venous thromboembolism is quite poor, so that D-dimer determination is only useful as an exclusionary test [26], [27]. In this assay, the D-dimer positive predictive value (PPV) was 0.70 and 0.65 for DVT and PE, respectively. In contrast, plasma SF cannot come from inflammatory sites, because of its high molecular weight. Thus the presence of SF in plasma is a marker of activation of *intravascular* coagulation.

We therefore developed an assay for SF determination based on D-dimer generated by the incubation of plasma with t-PA. After showing the specificity of the SF assay, the interest of determining SF level was evaluated in 119 patients with suspicion of DVT or PE. The cut-off value for SF, determined using ROC curves established from SF levels and an independent diagnosis (imagery) that classified the patients into two distinct groups (diseased and non-diseased) was 300 ng/mL.

The AUCs of the ROC curves (widely used for score performance) showed that SF is a better marker of thromboembolic disease than D-dimer (the AUCs for SF being 0.942 and 0.950 versus AUCs for D-dimer of 0.913 and 0.846 in DVT and PE, respectively). Calculated accuracy indices also showed that SF specificity for diagnosis of venous thromboembolism was much higher than that of D-dimer (SF specificity being 0.86 and 0.95 versus D-dimer specificity of 0.5 and 0.54 in DVT and PE, respectively). The positive predictive value of SF was also much higher than that of D-dimer for diagnosis of thromboembolic disease (the SF PPV being 0.89 and 0.94 versus a D-dimer PPV of 0.70 and 0.65 in DVT and PE, respectively).

The superior performance of the SF test as a predictor of thromboembolic disorders obviously depends on establishing appropriately standardized conditions for testing the samples. In particular, as described in the Materials and Methods, optimum incubation times and t-PA concentrations must be determined and respected. In addition, our lyophilized positive control was stable for several months, whereas SF levels measured in positive controls obtained by incubation of pooled normal plasma with varying amounts of thrombin (final concentration 0.08–0.2 IU/mL) for 2 min followed by thrombin blockage by adding heparin increased with time (results not shown), probably because fibrin monomer protects thrombin from inactivation by heparin-antithrombin III [28]]. Furthermore, in some cases the interpretation of SF levels for the diagnosis of thromboembolic disease should be undertaken with caution, because:

1) It has been shown that as soon as anticoagulants are administered, the SF level is normalized within a few hours. Usually, in patients with DVT or PE, anticoagulation with heparin is started as soon as possible to reduce the risk of an extension of the venous thromboembolic event, which, if left untreated, is often fatal or disabling. Consequently, in many patients with clinically suspected DVT or PE who are referred to a hospital, heparin therapy is started even before the diagnosis of thromboembolism is confirmed. Nevertheless, the SF analysis should be done only in patients not under anticoagulant therapy. This also explains why the daily variation of SF was not evaluated in our study.

2) In patients with a high basal level of D-dimer, since the SF value is calculated as the difference between the results of two D-dimer assays, it is possible that when the SF value is not very high, it could fluctuate around the cut-off value. But, in our cohort of patients, this was rarely observed: in suspected thromboembolic disease, we observed only two patients (one with PE and the other one with DVT) with a false negative SF level (20 and 60 ng/mL, respectively) accompanied by a very high level of D-dimer (3,860 and 2,440 ng/mL), and only one case of a false positive SF level (350 ng/mL) in a patient with suspected PE for whom the level of D-dimer was also high (3,210 ng/mL).

3) False-positive SF levels could arise from a very transient and moderate activation of coagulation or to pre-analytical conditions (activation during blood collection), leading to generation of SF in the plasma, but without reaching the stage of thrombus formation. Consequently, it is necessary to discard blood collected by a difficult venipuncture or without respecting the pre-analytical recommendations. Moreover, the false-negative SF levels seen in patients with D-dimer exceeding 500 ng/mL might also be due to a non-evolutive thrombotic process caused by a transient thrombin activity.

In the three thromboembolic patients with false negative D-dimer levels (one with DVT and two with PE), the measured SF concentrations exceed the upper limit of the normal cut-off value; therefore, it can be suggested that the false negative D-dimer values could be due to altered fibrin-clot structure (congenital or acquired), rendering the thrombi abnormally resistant to fibrinolysis [29]. The defective thrombolysis could directly contribute to the increased risk of thrombosis. This could explain the 3–5% false-negative D-dimer values in patients with confirmed DVT. Indeed, because of the high amount of t-PA added to the plasma to induce *in vitro* SF degradation, leading to D-dimer generation, and because SF is more easily degradable than are the tightly packed fibrin fibres in thrombi, SF degradation in vitro occurs even when there is abnormal resistance of fibrin clots to fibrinolysis.

Besides the interest of SF measurement for its positive predictive value in thromboembolic disorders, another advantage of this technique is that the SF level, like that of D-dimer, is expressed in fibrin equivalents, thereby enabling evaluation of the balance between coagulation activation and fibrin degradation. In contrast, the techniques using monoclonal antibodies reacting with epitopes located either in the fibrin-polymerization sites or in the recognition sites of enzymes that are the functional partners of fibrin, or else in conformational sites located in various regions of the alpha, beta and gamma chains [30]–[48], have consistently yielded different results depending on the monoclonal antibody used. Therefore, the use of monoclonal antibodies in this context is of limited utility. In conclusion**,** although the group of patients we were able to evaluate is too small to statistically test the value of concomitantly measuring D-dimer and SF levels in patients with suspected thromboembolism, the results are highly encouraging. We predict that concomitant determination of SF and D-dimer levels will prove to be clinically useful in the rapid diagnosis of thromboembolism. An added advantage to this strategy is that SF levels can potentially be used to monitor the effects of subsequent anticoagulant therapy.
